# Investigation of the underuse of adrenaline (epinephrine) and prognosis among patients with anaphylaxis at emergency department admission

**DOI:** 10.3389/fmed.2023.1163817

**Published:** 2023-07-07

**Authors:** Yen-Yue Lin, Hsin-An Chang, Yung-Hsi Kao, Chih-Pin Chuu, Wen-Fang Chiang, Ya-Chieh Chang, Yuan-Kuei Li, Chi-Ming Chu, Jenq-Shyong Chan, Po-Jen Hsiao

**Affiliations:** ^1^Department of Emergency, Taoyuan Armed Forces General Hospital, Taoyuan, Taiwan; ^2^Department of Emergency, Tri-Service General Hospital, National Defense Medical Center, Taipei, Taiwan; ^3^Department of Life Sciences, National Central University, Taoyuan, Taiwan; ^4^Department of Psychiatry, Tri-Service General Hospital, National Defense Medical Center, Taipei, Taiwan; ^5^Institute of Cellular and System Medicine, National Health Research Institutes, Zhunan, Taiwan; ^6^Graduate Program for Aging, China Medical University, Taichung, Taiwan; ^7^Division of Nephrology, Department of Internal Medicine, Taoyuan Armed Forces General Hospital, Taoyuan, Taiwan; ^8^Division of Nephrology, Department of Internal Medicine, Tri-Service General Hospital, National Defense Medical Center, Taipei, Taiwan; ^9^Division of Colorectal Surgery, Department of Surgery, Taoyuan Armed Forces General Hospital, Taoyuan, Taiwan; ^10^Department of Biomedical Sciences and Engineering, National Central University, Taoyuan, Taiwan; ^11^School of Public Health, National Defense Medical Center, Taipei, Taiwan; ^12^Graduate Institute of Life Sciences, National Defense Medical Center, Taipei, Taiwan; ^13^Graduate Institute of Medical Sciences, National Defense Medical Center, Taipei, Taiwan; ^14^Department of Public Health, School of Public Health, China Medical University, Taichung, Taiwan

**Keywords:** anaphylaxis, anaphylactic reactions, allergic reactions, adrenaline (epinephrine), emergency department

## Abstract

**Background:**

Anaphylaxis is a potentially fatal condition; in severe cases of anaphylaxis, the cardiovascular system is often heavily involved. Adrenaline (epinephrine) is a cornerstone of the initial treatment of anaphylaxis. The use of epinephrine remains below expectations in clinical practice. Whether the underuse of epinephrine affects the prognosis of patients with anaphylaxis is still unclear.

**Materials and methods:**

This retrospective study included patients with anaphylaxis between 2011 and 2020 who were admitted to an emergency department (ED) in Taiwan. All patients were divided into two groups based on the use of epinephrine (or not), and we compared the demographic characteristics, allergens, clinical manifestations, management, and patient outcomes.

**Results:**

We reviewed the records of 314 subjects (216 males, 98 females; mean age: 52.78 ± 16.02 years) who visited our ED due to anaphylaxis; 107 (34.1%) and 207 (65.9%) patients were categorized into the epinephrine use group and the non-epinephrine use group, respectively. Arrival via ambulance (*p =* 0.019), hypotension (*p* = 0.002), airway compromise (*p* < 0.001) and altered consciousness (*p* < 0.001) were the deciding factors for epinephrine use among anaphylactic patients in the ED. The epinephrine use group had higher rates of other inotropic agent usage and fluid challenge. More than 90% of patients received bed rest, steroids, antihistamines, and monitoring. The epinephrine use group had a longer ED length of stay (387.64 ± 374.71 vs. 313.06 ± 238.99 min, *p* = 0.03) and a greater need of hospitalization. Among all severe symptoms, hypotension was the most tolerated decision factor for not using epinephrine. In this retrospective analysis, some patients with serious anaphylaxis did not experience adverse outcomes or death even without the use of epinephrine at ED admission. Emergent care focuses first on the airway, breathing, and circulation (ABC) and may compensate for the underusage of epinephrine. This could be the reason why epinephrine was underused among patients with anaphylaxis in the ED.

**Conclusion:**

In summary, early ABC management continues to play an important role in treating patients with severe anaphylaxis, even when epinephrine is not immediately available in clinical scenarios.

## 1. Introduction

Anaphylaxis is considered a dramatic manifestation of systemic allergies. The prevalence of anaphylaxis is approximately 0.05%–2% in the USA and approximately 3% in Europe (1). Clinical cardiovascular manifestations include hypotension, shock, and sudden cardiac death caused by ventricular dysfunction, cardiac arrhythmias and cardiac arrest. The incidence of anaphylaxis in the emergency department (ED) has also increased both worldwide and in Taiwan (2–4). Adrenaline (epinephrine) remains the current first-line recommended treatment for anaphylactic reactions in all major guidelines throughout this period (5, 6). The delayed use of epinephrine may be associated with increased severity of reactions and fatalities (7). Although even guidelines and textbooks repeatedly reinforce use of this treatment, a literature review and clinical practice have revealed that the use of epinephrine for anaphylactic patients is very low (often less than 50%) (8, 9). The possible reasons for this may be a lack of physician knowledge about the recognition of anaphylaxis and fear of epinephrine-associated cardiovascular side effects (10, 11). Nevertheless, some patients with serious anaphylaxis do not have adverse outcomes or death even without the use of epinephrine (4, 12, 13). This research aimed to investigate the actual use of epinephrine and clinical outcomes among patients with anaphylaxis on admission to an ED in Taiwan.

## 2. Materials and methods

### 2.1. Study design and data collection

This was a 10 years descriptive retrospective analytical study. Medical records of inpatients and outpatients diagnosed with insect sting allergies at Taoyuan Armed Forces General Hospital from January 2011 to December 2020 were reviewed. Approximately 60,000 patients per year are admitted to the ED of this local teaching hospital. This study involved patients who met the inclusion criteria and were diagnosed with the International Statistical Classification of Diseases and Related Health Problems, 10th Revision codes (T780, T782, T805, T886 as ICD-10 codes). To date, the guidelines strongly recommend intramuscular epinephrine as the first-line treatment strategy (4–7). Two major groups, the epinephrine use group and the non-epinephrine use group, were included. Patients who received prehospital epinephrine, were aged <18 years, did not satisfy the above definition of anaphylaxis, or were transferred from or to other hospitals were excluded. Patients with incomplete data were also excluded.

### 2.2. Data collection and definition of the items

The patients’ clinical manifestations were divided into different systemic types, which were further classified as symptomatic and asymptomatic presentations according to the medical records. Additionally, the symptoms or signs for mild (such as only skin reaction) and severe anaphylaxis including noticeable cardiovascular manifestations (such as hypotension, chest pain, collapse), respiratory manifestations (such as upper airway obstruction, dyspnea, hypoxemia, wheezing, stridor) or altered consciousness were analysed. Low blood pressure was defined as a systolic pressure below 90 mmHg, cyanosis, or pulse oximetry saturation (SpO_2_) <92%.

The primary outcome was determined by the factors associated with the ED physicians’ decision to use epinephrine (or not). Our secondary outcomes were the ED stay, hospitalization, and mortality based on the use of epinephrine or not. Fluid challenge was defined as one to 2 litres of crystalloid fluid given rapidly within the first 2 h. For each patient, the following details were obtained from ambulance sheets, referral letters, and case notes: age, sex, comorbidities (asthma, chronic airflow limitation, hypertension, ischaemic heart disease, heart failure, previous stroke, or transient ischaemic attack), individual reaction features, likely reaction cause, and epinephrine administration.

### 2.3. Statistical analysis

Qualitative data are reported as percentages, and quantitative data are reported as the means or medians and minimum and maximum. Statistical analyses were carried out using SPSS version 16 (IBM, Armonk, NY). For intergroup comparisons, continuous data are expressed as the means ± standard deviations (SDs) and were tested with Student’s *t*-test. Categorical data are expressed as frequencies (%) and were tested with the chi-square test or Fisher’s exact test. To assess the adjusted effects of different variables, selected variables were used with a *p*-value <0.1 in the initial univariate results or clinically important factors for multivariate logistic regression analysis. Multivariate logistic regression analysis with forward stepwise selection was used to control for possible confounding variables and to determine the possible factors that influenced the use of epinephrine during ER stays between study groups. A *p*-value <0.05 was considered statistically significant.

## 3. Results

### 3.1. Patient enrolment and grouping

**Figure 1:**
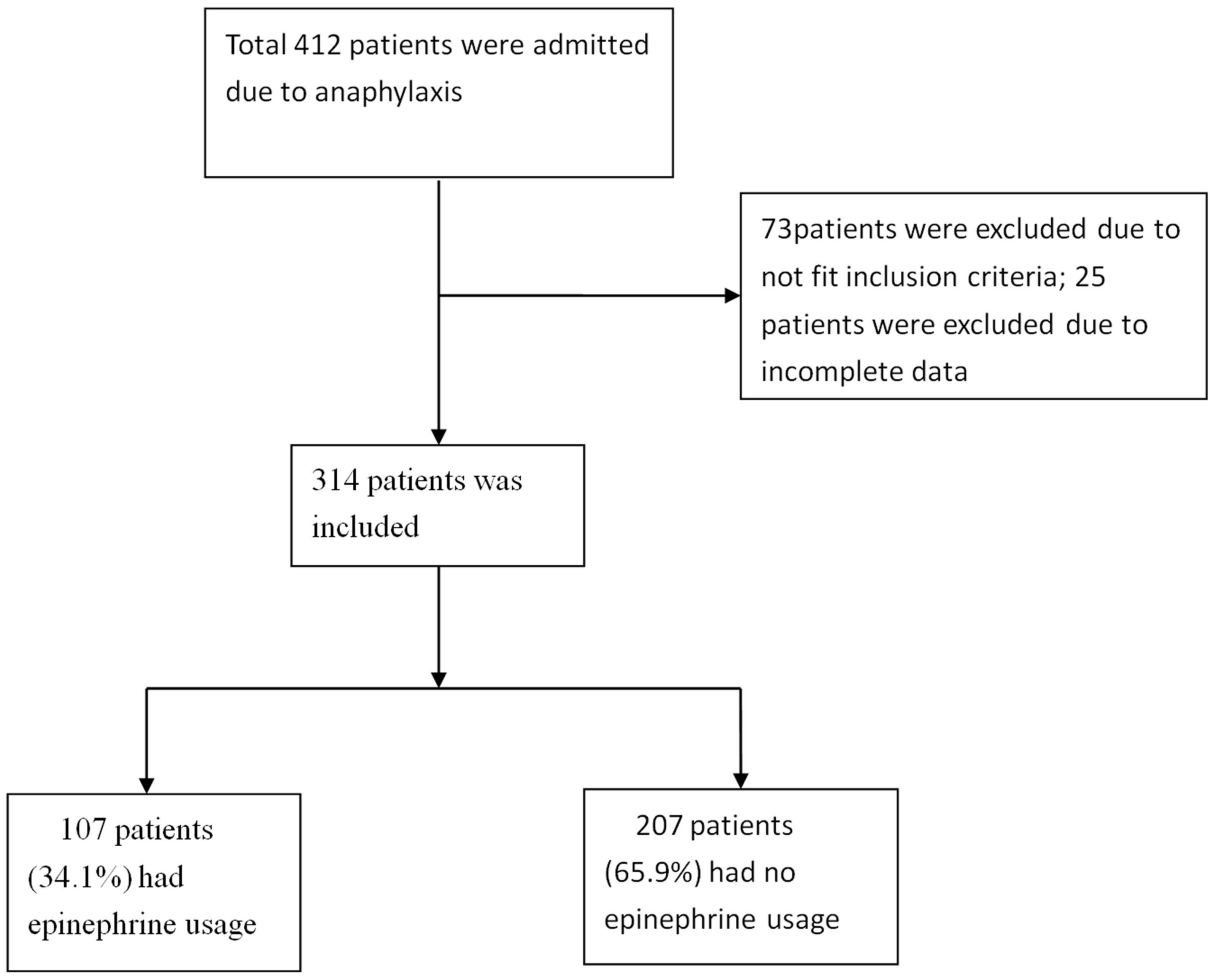
Flowchart of the participant inclusion process.

**Figure 2:**
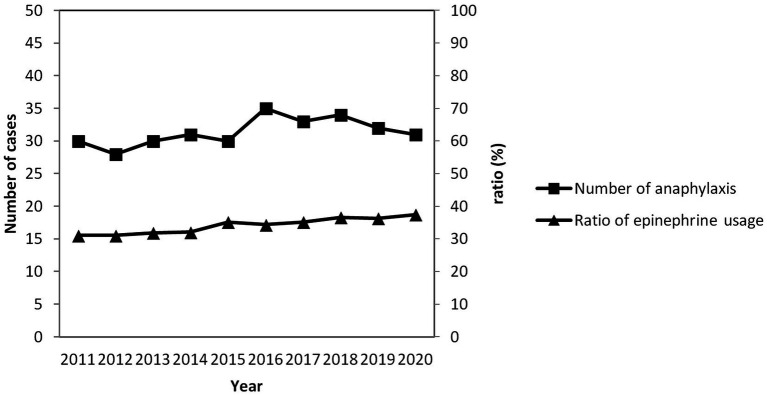
Proportion of ED patients who received epinephrine by year of anaphylaxis.

A total of 453 patient visits were coded with one of the ICD-10 codes related to anaphylaxis. Of these, 73 patients were excluded due to the prehospital usage of epinephrine (65 patients were given epinephrine by ambulance paramedics, and 8 patients used self-injectable epinephrine devices themselves), 51 patients were younger than 18 years, and 15 patients were excluded because they did not satisfy the definition of anaphylaxis. Of the remaining 314 study patients (216 men and 98 women; mean age, 52.78 ± 16.02 years), 107 (34.1%) were categorized into the epinephrine use group, and 207 (65.9%) were categorized into the non-epinephrine use group (Figure 1). The proportion of ED patients who received epinephrine by year of anaphylaxis was evaluated. The number of anaphylactic patients has slightly increased over the past decade. Although the proportion of epinephrine use also increased slightly, the highest was less than 40% (Figure 2). Patients who were sent via ambulance were more likely to be in the epinephrine group (83.2% vs. 67.0%, *p* < 0.01) (Table 1). Underlying comorbidities, allergy history, and trigger agents were not significantly different between the two groups.

**Table 1:** Demographic data of the study population.

Type of reaction	Total patients *n* = 314	Epi group *n* = 107 (34.1%)	Non-epi group *n* = 207 (65.9%)	*p*-value
Age (year)	52.78 ± 16.02	52.67 ± 16.99	52.83 ± 15.54	0.94
Male-no. (%)	216 (68.8)	71 (66.4)	145 (70.0)	0.50
Sent viaambulance-no. (%)	164 (52.2)	89 (83.2)	75 (36.2)	<0.01^*^
Comorbidities				
Hypertension-no. (%)	56 (17.8)	21 (19.6)	35 (16.9)	0.55
Diabetes-no. (%)^a^	27 (8.6)	8 (7.5)	19 (9.2)	0.68
Cardiac disease-no. (%)^a^	15 (4.8)	5 (4.7)	10 (4.8)	1.00
CVA-no. (%)^a^	3 (0.9)	0 (0)	3 (1.4)	0.55
CKD-no. (%)^a^	5 (1.6)	2 (1.9)	3 (1.4)	1.00
Asthma/COPD-no. (%)^a^	9 (2.9)	5 (4.7)	4 (1.9)	0.28
Gout-no. (%)^a^	8 (2.5)	2 (1.9)	6 (2.9)	0.72
Neoplasm-no. (%)^a^	2 (0.6)	1 (0.9)	1 (0.5)	1.00
Allergy history				
Drugs-no. (%)	53 (16.9)	22 (20.1)	31 (15.0)	0.21
Food-no. (%)^a^	24 (7.6)	10 (9.3)	14 (6.8)	0.50
Trigger				
Insect venom-no. (%)	124 (39.5)	36 (33.6)	88 (42.5)	0.07
Food-no. (%)	61 (19.4)	16 (15.0)	45 (21.7)	0.25
Drugs-no. (%)	85 (27.1)	33 (30.8)	52 (25.1)	0.24
Contrast media-no. (%)^a^	4 (1.3)	3 (2.8)	1 (0.5)	0.12
Contact-no. (%)^a^	6 (1.9)	4 (3.7)	2 (1.0)	0.19
Idiopathic-no. (%)^a^	34 (10.8)	15 (14.0)	19 (9.2)	0.25

Continuous data are expressed as the means ± SDs, and categorical data are expressed as numbers (%).

a Fisher’s exact test ^*^p < 0.05 CVA, cardiovascular accident; CAD, coronary artery disease; CKD, chronic kidney disease.

### 3.2. Comparison of the clinical presentation of both groups

Severe symptoms, such as hypotension (*p* = 0.03), airway compromise (*p* < 0.01), and altered consciousness (*p* < 0.01), were more frequently associated with the administration of epinephrine (Table 2).

**Table 2:** Clinical presentation of both groups.

Types of symptoms and signs	Total patients *n* = 314	Epi group *n* = 107 (34.1%)	Non-epi group *n* = 207 (65.9%)	*p*-value
Clinical presentation on arrival				
Cardiovascular				
Hypotension-no. (%)	142 (45.2)	61 (57.01)	81 (39.1)	0.03^*^
Chest pain-no. (%)^a^	30 (9.6)	11 (10.3)	19 (9.2)	0.75
Cutaneous				
Skin reaction-no. (%)	273 (88.4)	90 (84.1)	183 (88.4)	0.28
Angioedema-no. (%)^a^	14 (4.5)	6 (5.6)	8 (3.9)	0.57
Respiratory				
Respiratory-no. (%)	179 (57.0)	58 (54.2)	121 (58.5)	0.47
Airway compromise-no. (%)	50 (6.9)	48 (44.9)	2 (0.9)	<0.01^*^
Gastrointestinal				
N/V-no. (%)	47 (15.0)	13 (12.1)	34 (16.4)	0.31
Diarrhoea-no. (%)^a^	16 (5.1)	5 (4.7)	11 (5.3)	1.00
Neurological				
Altered consciousness-no. (%)	81 (25.8)	58 (54.2)	23 (11.1)	<0.01^*^
Dizziness-no. (%)^a^	23 (7.2)	9 (8.4)	14 (6.7)	0.60

Continuous data are expressed as the means ± SDs, and categorical data are expressed as numbers (%).

a Fisher’s exact test ^*^p < 0.05 h, hour; N/V, nausea/vomiting.

### 3.3. Comparison of treatment of both groups

Among the treatments used for the groups, other inotropic agent usage (*p* < 0.01) and fluid challenge (*p* < 0.01) were more frequent in the epinephrine use group. Only 34.1% of patients were treated with epinephrine, and 25.2% had delayed usage. More than 90% of patients were treated with steroids, antihistamines, and management with bed rest and monitoring (Table 3).

**Table 3:** Treatment for the two study groups.

Variable	Total patients (*n* = 314)	Epi group (*n* = 107)	Non-epi group (*n* = 204)	*p*-value
Medical treatment				
Epinephrine-no. (%)	107 (34.1)	107 (100)	0 (0)	–
Delayed use of epinephrine-no. (%)	27 (8.6)	27 (25.2)	0 (0)	–
Repeated use of epinephrine-no. (%)	18 (5.7)	12 (16.8)	0 (0)	–
Steroids-no. (%)	307 (97.8)	105 (98.1)	202 (97.6)	0.76
H1 blockers (antihistamines)-no. (%)	310 (98.7)	105 (98.1)	205 (99.0)	0.50
H2 blockers-no. (%)^a^	24 (7.6)	8 (7.5)	16 (7.7)	0.94
Beta-agonist nebulizer-no. (%)	51 (16.2)	14 (13.1)	37 (17.9)	0.28
Other inotropic agents-no. (%)^a^	13 (4.1)	11 (10.2)	2 (0.9)	<0.01^*^
ED supportive care				
O_2_ supplementation-no. (%)^a^				
Intubation-no. (%)^a^	2 (0.6)	2 (1.9)	0 (0.0)	0.12
Fluid challenge-no. (%)	156 (49.7)	69 (64.5)	87 (42.0)	<0.01^*^
Bed rest-no. (%)	299 (95.2)	103 (96.3)	196 (94.7)	0.54
Monitoring-no. (%)	284 (90.5)	98 (91.6)	186 (89.9)	0.62

IV, intravenous; IM, intramuscular. Data are expressed as numbers (%).

a Fisher’s exact test ^*^p < 0.05.

### 3.4. Logistic regression analysis of factors associated with epinephrine use for anaphylactic patients in the ED

Logistic regression analysis was conducted to compare the study groups. Arrival via ambulance (*p* = 0.019), hypotension (*p* = 0.002), airway compromise (*p* < 0.001), and altered consciousness (*p* < 0.001) were deciding factors for epinephrine use for anaphylactic patients in the ED (Table 4).

**Table 4:** Logistic regression analysis for decision factors associated with epinephrine use for anaphylactic patients in the ED.

Variable	Univariate regression analysis		Multivariate regression analysis	
	OR (95% CI)	*p*-value	OR (95% CI)	*p*-value
Age	1.00 (0.99–1.02)	0.94	0.99 (0.97–1.02)	0.914
Sex				
Female	1.00	–	1.00	–
Male	0.84 (0.51–1.39)	0.503	0.68 (0.31–1.47)	0.324
Sent via ambulance				
No	1.00	–	1.00	–
Yes	2.36 (1.32–4.24)	0.004^*^	2.97 (1.20–7.33)	0.019^*^
Insect sting				
No	1.00	–	1.00	–
Yes	0.63 (0.39–1.03)	0.066	0.72 (0.33–1.56)	0.404
Hypotension				
No	1.00	–	1.00	–
Yes	2.06 (1.28–3.31)	0.003^*^	3.49 (1.61–7.56)	0.002^*^
Airway compromise				
No	1.00	–	1.00	–
Yes	83.39 (19.68–353.31)	<0.001^*^	284.74 (55.39–1463.63)	<0.001^*^
Change in consciousness				
No	1.00	–	1.00	–
Yes	9.47 (5.32–16.86)	<0.001^*^	17.12 (8.01–36.57)	<0.001^*^

OR, odds ratio; CI, confidence interval, ^*^p < 0.05.

### 3.5. Percentage of epinephrine use in terms of decision factors

**Figure 3:**
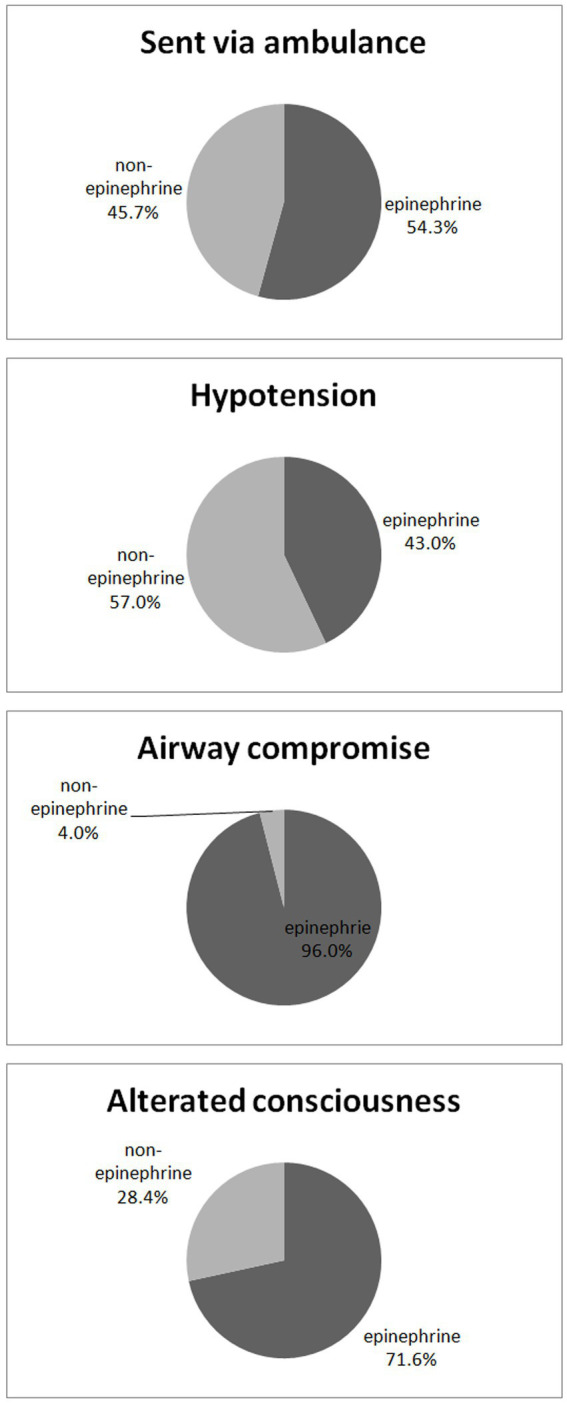
Percentage of epinephrine use in terms of significant decision factors.

Fewer than 50% of patients with hypotension (among all significant decision factors) received epinephrine treatment (Figure 3).

### 3.6. Results for the treatment population

**Figure 4:**
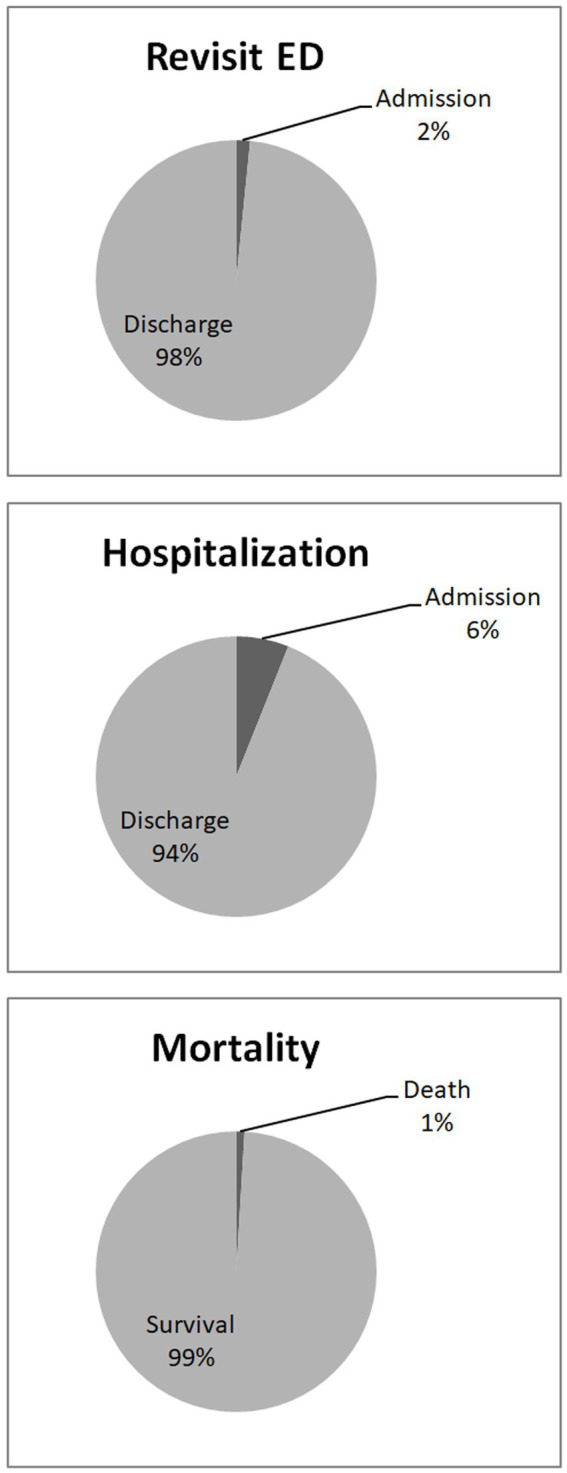
Proportions of patient outcomes.

The patients in the epinephrine use group had a longer length of ED stay (*p* < 0.01) and a higher rate of need for hospitalization (*p* = 0.03) (Table 5). The proportions of patients that experienced ER recall, hospitalization, and mortality were very low (Figure 4).

**Table 5:** The ED length of stay, revisit, mortality, and hospitalization in the both two groups.

	Total patients *n* = 314	Epi group *n* = 107 (34.1%)	Non-epi group *n* = 207 (65.9%)	*p*-value
ED length of stay (minutes)	338.47 ± 293.93	387.64 ± 374.71	313.06 ± 238.99	0.03^*^
ED revisit-no. (%)^a^	**5 (0.01)**	**1 (0.03)**	**4 (0.0)**	**0.26**
Mortality-no. (%)^a^	3 (0.01)	3 (0.03)	0 (0.0)	0.26
Hospitalization-no. (%)^a^	19 (6.1)	14 (13.1)	5 (2.4)	<0.01^*^

Continuous data are expressed as the means ± SDs, and categorical data are expressed as numbers (%).

a Fisher’s exact test ^*^p < 0.05 h, hour.

### 3.7. Statistics for the reasons for mortality and hospitalization

A total of 19 patients (mostly in the epinephrine use group) were admitted due to multiple complications (the most common was acute coronary syndrome, including myocardial infarction) and 3 patients died. Only 2 patients in the non-epinephrine use group were admitted (Table 6).

**Table 6:** Case numbers for the reasons for mortality and hospitalization in the both two groups.

Variable	Total number of anaphylactic patients	Epi group	Non-epi group
Mortality (total number = 3)			
Unsuccessful resuscitation	1	1	0
MODS^§^	2	2	0
Hospitalization (total number = 19)			
Acute coronary syndrome	3	3 (2 MIs)	0
Ischaemic stroke	1	1	0
Rhabdomyolysis	4	3	1
Acute renal failure	4	3	1
MODS^§^	2	2	0
Unsubsidized allergy	5	5	0

MODS, multiple organ dysfunction syndrome; MI, myocardial infarction, § = 3 cases of mortality after hospitalization.

## 4. Discussion

This is the first study conducted in the ED to investigate why there is a low rate of epinephrine use and low mortality among anaphylactic patients in Taiwan. This study found that ED doctors tend to use epinephrine for anaphylactic patients with severe symptoms, such as hypotension, airway compromise and altered consciousness, and for anaphylactic patients who arrive via ambulance. Patients with hypotension had the lowest rate of epinephrine usage. Supplemental treatment could compensate for the underuse of epinephrine for anaphylaxis and lower the mortality rate.

### 4.1. Causes and diagnosis of anaphylaxis

#### 4.1.1. Aetiologies and mechanisms of anaphylaxis

It has been reported that the estimated prevalence of anaphylaxis is approximately 0.05%–2% in the USA and 3% in Europe across the lifetime of the patient (14, 15). Most anaphylactic episodes involve the immunologic mechanism of immunoglobulin E (IgE) reactions. The traditional pathway of anaphylaxis is facilitated through T cells, Th2 cytokines, B-cell production of IgE and consequent crosslinking of the high-affinity IgE receptor (FcεRI) on mast cells and basophils by IgE-antigen complexes, terminating in mast cell and basophil degranulation (1–3). Degranulation further causes the production of mediators [histamine, chymase, tryptase, heparin, cathepsin G, carboxypeptidase, and tumour necrosis factor alpha (TNF-α)] and of *de novo* synthesized mediators, including lipid mediators platelet-activating factor (PAF), cysteinyl leukotrienes, cytokines and growth factors such as vascular endothelial growth factor (VEGF). Of these, histamine, tryptase, cathepsin G, TNF-α, LTC4, PAF and VEGF can increase vascular permeability (3–5). Foods are the most common trigger object in children, while medications and insect stings are more common in adults (1). In our study, insects were the most common cause of anaphylaxis, which may be due to our hospital being located near a rural area. Hornet and fire ant stings were the two most common insect stings, especially in the summer period (8). Contrast allergy is low for nonionic and much lower for ionic contrast agents (16). Our hospital routinely used a nonionic contrast medium for all patients during the study period, so there was a rare case with an anaphylactic reaction.

#### 4.1.2. Definition of anaphylaxis

The definitions of anaphylaxis vary (17). Anaphylaxis can be simply defined as a severe allergic reaction that involves more than one organ system (18). Some definitions focus on the rapidity of onset with potentially life-threatening problems such as allergic reactions (19). In recent years, the diagnostic criteria from the second National Institute of Allergy and Infectious Diseases/Food Allergy and Anaphylaxis Network (NIAID/FAAN) symposium were the most widely utilized and were reported to have around 95% sensitivity for the diagnosis of anaphylaxis (18, 20). There is no universal consensus on the diagnosis of anaphylaxis (21), and therefore, ED physicians may reach different diagnoses for this allergic reaction. Recently, Dribin et al. (22) recommended a new severity grading system for the acute allergic reactions including the non-anaphylactic and anaphylactic reactions. They also demonstrated a successful international validation and application in this grading system. The grading system could improve a communication between the providers and patients about the severity of allergic reactions. Their research is the first report to perform a consensus-based severity grading system for acute allergic reactions based on the use of Delphi methodology, which may be a preferred and broadly used methodology in the future. Although the use of this severity grading system may help clinicians to accurately assess the severity of anaphylaxis and standardise management, it has not been adopted in EDs worldwide. The patients included in our study would have had different severities; some may have been overdiagnosed, and some may have been underdiagnosed.

#### 4.1.3. The diagnosis of anaphylaxis

The recognition of anaphylaxis can be difficult without cutaneous manifestations. One study reported that up to 20% of cases may have no reaction or a subtle skin reaction, and some cases would thus be treated as asthma or shock with an unknown cause (19). This may lead to delayed recognition and a low rate of epinephrine use. In our study, we found that most of the doctors used diagnostic criteria that were not very precise; most of the physicians used the two-system criteria.

#### 4.1.4. Comorbidities

Associated comorbidities and medications may play a role in the severity of anaphylactic reactions and patient responses to treatment. Patients with asthma and cardiovascular disease are more likely to experience a poor outcome from anaphylaxis. These comorbidities may impact the severity of anaphylaxis. Concurrent administration of beta blockers can interfere with the patient’s ability to respond to epinephrine (23). In middle-aged and older patients, cardiovascular disease is an important risk factor for death from anaphylaxis (24). The use of angiotensin-converting enzyme (ACE) inhibitors may impact a patient’s compensatory physiologic response to anaphylaxis, resulting in more severe reactions, although the evidence is conflicting (6, 23). Recent research indicates that the use of any antihypertensive medication may deteriorate an anaphylactic reaction (25). However, we did not find significance for this in our study. The number of patients may have been low, and there were fewer older patients in our study.

### 4.2. Clinical management in the ED

Anaphylaxis treatment begins with a rapid assessment and maintenance of the airway, breathing, and circulation. The cornerstones of the treatment are immediate discontinuation of the trigger and early epinephrine administration (23). Otherwise, other medications and procedures are also helpful.

#### 4.2.1. Medication management

##### 4.2.1.1. Epinephrine

Epinephrine is the first and most important treatment for anaphylaxis (11, 26). Most of the literature emphasizes that all patients with anaphylaxis must be appropriately treated with intramuscular epinephrine (27). There are no absolute contraindications to epinephrine use for anaphylaxis (11, 23). In fact, not administering epinephrine when needed would be the most serious safety problem in the management of patients with anaphylaxis.

The preferred route for the initial administration of epinephrine for anaphylaxis is the intramuscular (IM) injection route for most settings and patients of all ages (28, 29). IV bolus epinephrine should be avoided because it is associated with substantially more dosing errors and cardiovascular complications than IM epinephrine (11, 30). If the anaphylaxis dose does not respond to intramuscular epinephrine and intravenous fluid challenge, an intravenous infusion of epinephrine may be needed. As the severity of anaphylaxis increases, some patients may require more than 1 dose of epinephrine (31).Recently, the use of rapidly disintegrating sublingual epinephrine tablets was reported to be useful as an easy-to-carry, palatable, noninvasive treatment for severe anaphylactic reactions in community settings (32).

The administration of epinephrine in therapeutic doses may result in some transient pharmacologic effects, including restlessness, headache, dizziness, tremor, palpitations, anxiety, and pallor (11, 33). Angina, myocardial infarction, ventricular arrhythmias, pulmonary oedema, a sudden sharp increase in blood pressure, and intracranial haemorrhage may rarely occur after the use of epinephrine, although anaphylaxis itself can result in the above cardiovascular complications in the absence of any exogenous epinephrine or before exogenous epinephrine is administered (34). Therefore, the use of epinephrine also has several adverse effects that range from mild to severe (11). Serious adverse effects can occur after an IV bolus injection, particularly if an inappropriately large dose is administered (11, 28). Some patients in our study were admitted for acute coronary syndrome, and epinephrine use could not be ruled out as a cause.

##### 4.2.1.2. H1-antihistamines

Despite the lack of strong evidence and guideline recommendations supporting their use for anaphylaxis, antihistamines are commonly used to treat such patients (35). Antihistamines can also be helpful for the control of cutaneous symptoms, but they do not relieve upper or lower airway obstruction, hypotension, or shock. Moreover, they do not inhibit mediator release from mast cells and basophils at standard doses (23, 36). Antihistamines should never replace epinephrine as first-line therapy. In our study, there was also a high percentage of antihistamine and steroid use. One reason is that a high percentage of anaphylactic patients have cutaneous symptoms, and another reason is that doctors may habitually prescribe an antihistamine to any disease associated with an allergic problem.

##### 4.2.1.3. Bronchodilators

For the management of bronchospasm, inhaled bronchodilators (e.g., albuterol, salbutamol) should be administered by a mouthpiece and nebulizer/compressor. Patients with milder respiratory symptoms can receive albuterol by a metred-dose inhaler. Bronchodilators are an adjunct treatment because they do not prevent or relieve upper airway mucosal oedema or shock, for which the alpha-1-adrenergic effects of epinephrine are needed (21, 23–26). The evidence for the use of beta-2-adrenergic agonists in anaphylaxis is extrapolated from their use in acute asthma.

##### 4.2.1.4. Corticosteroids

To date, there is no recommendation that glucocorticoids should be used for the treatment of anaphylaxis (37). Corticosteroid effects have a slow onset and are not effective for the acute treatment of anaphylaxis (23). Theoretically, they may prevent biphasic or protracted reactions and, hence, are often given on an empirical basis. Although there is no strong evidence that the administration of corticosteroids prevents a biphasic response, a recent nonrandomized study suggested otherwise (38). There was a high percentage of steroid use in the two study groups and a low rate of a biphasic reaction. Despite the lack of strong evidence, ED doctors tend to routinely use steroids for anaphylactic patients.

##### 4.2.1.5. Inotropic agents

Vasopressors such as dopamine and norepinephrine should be considered if epinephrine injections and volume expansion with intravenous fluids fail to alleviate hypotension. The addition of another vasopressor should be considered if the patient continues to be hypotensive despite maximal epinephrine and fluid therapy. It is not well known whether the addition of other vasopressors is superior to epinephrine alone, but one theory about the pathogenesis of refractory anaphylaxis proposes that the clinical manifestations may become refractory to further catecholamine administration, perhaps due to saturation or desensitization of adrenergic receptors (39). The use of vasopressin in the management of anaphylaxis refractory to intravenous (IV) epinephrine can also be considered (40). In our study, the use of inotropic agents was significantly greater in the epinephrine use group. This suggested that the epinephrine use group had a more severe overall condition.

#### 4.2.2. Non-medication management

##### 4.2.2.1. ED supportive care and monitoring

In addition to epinephrine administration, the most important step is a rapid assessment of circulation and breathing (41). Vital signs (blood pressure, heart rate, and oxygenation) should be monitored continuously or as soon as possible. Supplemental oxygen and intravenous fluid could be administered, and if necessary, cardiopulmonary resuscitation should be performed (42). This management is compatible with the basic principles of ED management and ACLS guidelines. Taiwan has already been utilizing an emergency specialty for more than 20 years (43). Experienced ED physicians could also play a good role in life support treatment. Therefore, a similar process may result in low complication and mortality rates when managing anaphylactic patients in the ED.

##### 4.2.2.2. Airway management

Anaphylaxis-related angioedema is a serious disorder that can lead to fatal airway obstruction, which should immediately be treated with intubation and mechanical ventilation (42, 44, 45). Early recognition of the desaturation of a patient is mandatory. Epinephrine use could decrease angioedema and asthmatic symptoms in anaphylactic patients and reduce the need for intubation. In our study, 5 anaphylactic patients were intubated in the ED. The proper use of epinephrine in severe cases may be the main reason for this observation.

##### 4.2.2.3. Oxygen therapy and intubation

Oxygen therapy should also be considered for any patient with symptoms of anaphylaxis, particularly for those with prolonged reactions and cardiovascular or pulmonary reactions (23). Only in rare cases will endotracheal intubation be needed, and it is recommended that the procedure be performed by an experienced physician. Oedematous airways may become a problem for intubation (46).

##### 4.2.2.4. Posture

Since an upright posture may be a feature of fatal anaphylaxis, to prevent distributive shock and empty vena cava/empty ventricle syndrome, a patient who is diagnosed with anaphylaxis should be placed in the supine position with the lower extremities elevated unless there is prominent severe upper airway swelling (13, 47). Alternate postures, such as the Trendelenburg position and passive leg raise (which are thought to increase cardiac output and elevate blood pressure), are used to treat hypotension (48, 49). If vomiting occurs, placement of the patient in the semirecumbent position with the lower extremities elevated may be preferable. Pregnant patients should be placed on their left side (42). Patients in our study were treated with bed rest, which may have compensated for the symptoms of hypotension even without epinephrine use.

##### 4.2.2.5. Volume replacement

Volume replacement is especially important for anaphylaxis patients with persistent hypotension despite epinephrine injections. Intravenous (IV) access should be achieved in all cases of anaphylaxis. Intravenous crystalloid solutions should also be provided because massive fluid shifts caused by increased vascular permeability can develop quickly, with the transfer of up to 35% of the intravascular volume into the extravascular space within minutes (50). Any patient whose hypotension does not respond promptly and completely to IM epinephrine should receive a large volume of fluid resuscitation (23, 51).

##### 4.2.2.6. Monitoring

Anaphylactic patients should be observed in a medical facility and are recommended to be monitored for late-phase reactions, although these rarely occur (52). The delayed detection of patient deterioration often results in an increased length of stay in the intensive care unit and poor patient outcomes (53, 54). Current evidence shows that early signs of deterioration can be predicted by the patient’s vital sign changes (55). More than 90% of patients with anaphylaxis in our study received monitoring, including blood pressure, respiratory rate, and O_2_ saturation measurements.

### 4.3. Delayed use of epinephrine due to misdiagnosis with similar diseases

Although consensus guidelines recommend epinephrine as first-line therapy, many physicians still tend to administer antihistamines and corticosteroids as the first medications for anaphylactic patients (56, 57). In our study, more than 90% of anaphylactic patients received antihistamines and corticosteroids. Some studies have indicated that different reaction patterns may cause clinical uncertainty and are attributed to delayed epinephrine usage, such as an initial misdiagnosis of shock or an asthma attack (7, 19). However, we found in our study that this may not be a serious problem. We found similar management of asthmatic symptoms in anaphylaxis. Standard asthma treatments also include beta-adrenergic agonists and steroids (58). Epinephrine is used in life-threatening situations (45, 59). The principle of shock patient management focuses on fluid supplements and vasoactive agent usage; in extreme situations, epinephrine is also used.

#### 4.3.1. Comparison of anaphylaxis and asthma management

Asthma and anaphylaxis are both allergic diseases, and they share many similar mechanisms. A severe asthma attack may lead to diagnostic confusion because wheezing, coughing, and shortness of breath can occur in both clinical conditions; however, skin itching, rash, urticaria, angioedema, abdominal pain, and hypotension are unlikely in patients with an acute asthma attack (60). Asthma and anaphylaxis are both hypersensitive conditions that can be either allergic or nonallergic (61). Many of their medications and management strategies are also similar, such as beta-2 agonists, steroids, and epinephrine, which are also suggested for use in serious conditions (62). We found that beta-2 agonists were often used for our study patients to treat breathing problems, and almost all patients received steroids.

#### 4.3.2. Comparison of anaphylaxis and shock management

Anaphylaxis is categorized as a distributive shock. It is an immunoglobulin E-related hypersensitivity reaction in which the release of bioactive factors causes vasodilation, leading to hypotensive shock (60). The general approach to shock is to secure basic life support. The treatment of distributive shock involves the combination of vasoactive medications and fluid challenge. Vasoactive medications can constrict the dilated vasculature, and fluids can fill the expanded vascular volume. Organ perfusion, however, is preserved, and tolerating hypotension may be an optional choice (63). Hypotension is a relatively benign condition that is underrecognized mainly because it is typically asymptomatic (64). Permissive hypotension is commonly employed in the trauma setting for patients experiencing acute haemorrhagic volume depletion (65). Our study showed that without a severe airway or hypoxic status and change in consciousness, permissive hypotension without epinephrine use but with the adoption of another way to resuscitate the patient and to avoid epinephrine use seems to be safe.

### 4.4. Disposition

#### 4.4.1. Observation

All patients with anaphylaxis should be observed in the ED until their symptoms have completely resolved. Some papers have suggested that patients should be observed for at least 4–6 h, but there is still no consensus on the optimal observation period (19, 52, 66, 67). A recent guideline suggested that patients with complete resolution of symptoms and no high-risk features may be discharged safely after 1 h of observation (66). The major concern after discharge is the biphasic reaction. Recent studies, however, have reported that clinically significant biphasic reactions in anaphylaxis are quite rare (68–70). We observed a recall of anaphylactic patients of less than 0.5% due to an allergy-related problem. We also observed in our study that patients treated with epinephrine spent more time in the ED. This may not mean that epinephrine use could not shorten the time of patient stay in the ED, but it reflected the fact that ED physicians used epinephrine for many patients with severe anaphylaxis.

#### 4.4.2. Hospitalization

The severity of symptoms and the receipt of multiple doses of epinephrine were associated with hospitalization. Patients with a higher risk for mortality (e.g., cardiovascular comorbidities, poor self-care, lack of access to emergency medical services, lack of access to epinephrine), multiple Vespidae stings, complications of other diseases, and an extended observation period were also recommended for admission (8, 66, 71). Similar to the ED length of stay, almost all patients who needed admission in our study received epinephrine. This could also be the reason that ED physicians tended to use epinephrine in more severe cases. However, less than 50% of patients did not directly exhibit allergic problems but, rather, the complications of other diseases, such as acute coronary syndrome, ischaemic stroke, rhabdomyolysis, and renal failures. Most allergic problems could be solved with ED management, but the need for admission was low.

#### 4.4.3. Mortality

Death is the most serious complication of anaphylaxis. Current guidelines indicate that anaphylaxis can be a “life-threatening reaction,” but in general, mortality and morbidity do not seem to have increased in recent decades (56). One study indicated that approximately one-half of deaths occur within 1 h after the onset of anaphylaxis (24). The failure to recognize the severity of the reactions and to administer epinephrine in a timely manner may promptly increase the risk of a fatal outcome (72). The rate of mortality of anaphylaxis is rare, and the total mortality risk of anaphylaxis constitutes less than 1% (13). This finding is similar to those of several recent studies, and the major causes of death in anaphylaxis usually result from upper or lower airway obstruction or cardiovascular collapse (7, 9, 73, 74). The risk factors for fatal anaphylaxis may vary based on the original cause. For fatal drug anaphylaxis, previous cardiovascular morbidity and older age are risk factors, with beta-lactam antibiotics, general anaesthetic agents, and radiocontrast injections being the most common triggers. For fatal food anaphylaxis, delayed administration of epinephrine is a risk factor; common triggers are nuts, seafood, and milk for children. For fatal venom anaphylaxis, risk factors include middle age, male sex, white race, cardiovascular disease, and possibly mastocytosis; insect triggers vary by region. Upright posture is a feature of fatal anaphylactic reactions to both food and venom (13). In our study, there was no mortality during the emergency room visit, and four patients died after admission due to multiple organ failure for multiple hornet stings. Emergency management seemed to strongly secure the survival of patients with anaphylaxis in the ED, even with the low rate of epinephrine usage. However, the low mortality rate may reduce ED doctor compliance and lead them to ignore the importance of the use of epinephrine as a recommended first-line management.

The present research design was that of a nonrandomized observational retrospective study with a small study population and routinely recorded patient ED data, and ED diagnoses and treatments were not standardized. Because the definition of anaphylaxis has not yet been settled, it may influence the ED physician’s coding with the ICD system. The recognition of the cause or precise symptoms was not accurate because this information was based on the patient’s memory (recall bias) or discrimination. Some medical information may be missing; for example, the patients had forgotten some important information at the ED admission. It was difficult to identify the real severity of anaphylaxis symptoms by consulting only the medical records. Another limitation of this study is that the diagnosis of anaphylaxis was made by various doctors, each of whom had different judgements on their respective patients. Some patients with relatively mild symptoms were also included. The present study was performed in Taiwan and only included adult patients; therefore, the results may differ from those in other populations worldwide. Not all symptoms and signs of anaphylaxis occur simultaneously. Many serious anaphylactic reactions occur quickly before patient arrival at the ED, and most anaphylactic patients may not be in serious conditions when they arrive at the ED. We already excluded those patients with prehospital epinephrine usage, and this may also have excluded more serious patients. However, there were still many patients with very serious symptoms with prehospital epinephrine use, and they experienced no ominous outcomes or death after ED treatment. Some symptoms may have occurred during the ED visit. The biphasic reaction may not have been recorded completely if the symptoms were not carefully observed or were omitted as part of normal clinical practice. Although most patients were placed under surveillance by the monitoring of vital signs, not all serious, poor vital signs would be recorded in the chart unless the patients were symptomatic or had sufficiently severe anaphylaxis. Finally, our retrospective design may lack the use of severity score and the quantifying clinical parameters such as blood pressure, heart rate, oxygen saturation, IgE and other biochemical markers.

## 5. Conclusion

Anaphylaxis can be a potentially fatal and multisystem syndrome in the absence of appropriate and timely treatment. Epinephrine should still be emphasized for use in anaphylaxis with severe symptoms. Modern ED management may already play a good role in managing anaphylaxis. Epinephrine may be safely preserved for anaphylaxis patients with severe symptoms, such as airway compromise and altered consciousness, according to the ED physician’s clinical decision. In conclusion, timely and appropriate airway, breathing, and circulation management continues to play a major role in treating emergency anaphylaxis patients, especially when epinephrine is not immediately obtainable.

## Data availability statement

The datasets generated and analysed during the current study are available from the corresponding author upon reasonable request.

## Ethics statement

The studies involving human participants were reviewed and approved by the institutional review boards of the Tri-service General Hospital (TSGH-IRB-A202105064). Written informed consent for participation was not required for this study in accordance with the national legislation and the institutional requirements.

## Author contributions

P-JH, Y-YL, Y-HK, C-PC, H-AC, Y-CC, Y-KL, and J-SC: conceptualization. P-JH, H-AC, and Y-YL: methodology. P-JH, Y-YL, and W-FC: validation. P-JH, Y-YL, and C-MC: investigation. P-JH and Y-YL: data curation, writing—review and editing, and funding acquisition. Y-YL: writing—original draft preparation. C-PC and J-SC: supervision. P-JH, C-PC, Y-HK, and Y-YL: project administration. All authors contributed to the article and approved the submitted version.

## Funding

This study was supported in part by grants from the Taoyuan Armed Forces General Hospital (TYAFGH-D-111024, TYAFGH-D-112025, and TYAFGH-A-112013).

## Conflict of interest

The authors declare that the research was conducted in the absence of any commercial or financial relationships that could be construed as a potential conflict of interest.

## Publisher’s note

All claims expressed in this article are solely those of the authors and do not necessarily represent those of their affiliated organizations, or those of the publisher, the editors and the reviewers. Any product that may be evaluated in this article, or claim that may be made by its manufacturer, is not guaranteed or endorsed by the publisher.
